# Identification and characterization of alternative splicing in parasitic nematode transcriptomes

**DOI:** 10.1186/1756-3305-7-151

**Published:** 2014-04-01

**Authors:** Sahar Abubucker, Samantha N McNulty, Bruce A Rosa, Makedonka Mitreva

**Affiliations:** 1 The Genome Institute, Washington University School of Medicine, 4444 Forest Park Boulevard, St. Louis, MO 63108, USA; 2 Division of Infectious Diseases, Department of Internal Medicine, Washington University School of Medicine, St. Louis, MO 63110, USA; 3 Department of Genetics, Washington University School of Medicine, St. Louis, MO 63108, USA

**Keywords:** Parasitic nematodes, Transcriptomes, Alternative splicing, Next-generation sequencing

## Abstract

**Background:**

Alternative splicing (AS) of mRNA is a vital mechanism for enhancing genomic complexity in eukaryotes. Spliced isoforms of the same gene can have diverse molecular and biological functions and are often differentially expressed across various tissues, times, and conditions. Thus, AS has important implications in the study of parasitic nematodes with complex life cycles. Transcriptomic datasets are available from many species, but data must be revisited with splice-aware assembly protocols to facilitate the study of AS in helminthes.

**Methods:**

We sequenced cDNA from the model worm *Caenorhabditis elegans* using 454/Roche technology for use as an experimental dataset. Reads were assembled with Newbler software, invoking the cDNA option. Several combinations of parameters were tested and assembled transcripts were verified by comparison with previously reported *C. elegans* genes and transcript isoforms and with Illumina RNAseq data.

**Results:**

Thoughtful adjustment of program parameters increased the percentage of assembled transcripts that matched known *C. elegans* sequences, decreased mis-assembly rates (i.e., cis- and trans-chimeras), and improved the coverage of the geneset. The optimized protocol was used to update *de novo* transcriptome assemblies from nine parasitic nematode species, including important pathogens of humans and domestic animals. Our assemblies indicated AS rates in the range of 20-30%, typically with 2-3 transcripts per AS locus, depending on the species. Transcript isoforms from the nine species were translated and searched for similarity to known proteins and functional domains. Some 21 InterPro domains, including several involved in nucleotide and chromatin binding, were statistically correlated with AS genetic loci. In most cases, the Roche/454 data explored in this study are the only sequences available from the species in question; however, the recently published genome of the human hookworm *Necator americanus* provided an additional opportunity to validate our results.

**Conclusions:**

Our optimized assembly parameters facilitated the first survey of AS among parasitic nematodes. The nine transcriptome assemblies, their protein translations, and basic annotations are available from Nematode.net as a resource for the research community. These should be useful for studies of specific genes and gene families of interest as well as for curating draft genome assemblies as they become available.

## Background

Alternative splicing (AS) is a post-transcriptional, mRNA modification process that allows a single gene to give rise to multiple protein isoforms [1,2]. These spliced isoforms can have distinct molecular functions and biological roles and may be differentially expressed among tissues, life cycle stages or environmental conditions [3], resulting in involvement in more genetic interactions and biochemical pathways compared to non-AS genes [4]. Therefore, AS provides a significant boost to genomic complexity without necessitating a proportional increase in genome size. AS takes place to some extent in most eukaryotic organisms [5-7], and has been studied extensively in humans and model species, including *Caenorhabditis elegans*[8-12], but has not received much attention in parasitic nematodes. In fact, information on AS in parasitic nematodes is extremely sparse, and existing reports have focused on a few or single representative gene(s) [13-16].

High throughput cDNA sequencing is the preferred method for detecting and quantifying AS. Today’s most prevalent sequencing protocols (e.g., 454, Illumina, Ion Torrent, etc.) involve fragmentation of nucleic acid molecules, construction of sequencing libraries, and generation of many thousands to millions or even billions of short reads. In the absence of a well-curated genome for comparison, these reads must be re-assembled *de novo* into contiguous sequences (contigs) that faithfully represent the full-length transcripts from which they were derived. Graph-based assembly algorithms have been developed to maintain associations between transcripts with shared contigs, making it possible to identify different isoforms of the same gene [17]. However, this procedure is computationally challenging, and various studies have shown that *de novo* cDNA assemblers typically overestimate the number of isoforms associated with a given locus and that many of the predicted isoforms are illegitimate [18-21]. Care must be taken to optimize assembly parameters to minimize errors and maximize accuracy and coverage.

cDNA sequencing is a cost-effective means of gene discovery in non-model organisms, so it often serves as the first line of investigation into an organism’s genetic complement. Thus, the transcriptomes of many parasitic nematodes (often including multiple sexes and life cycle stages) have been sequenced and relevant datasets are readily available [22]. Several *de novo* transcriptome assemblies have been reported [23-30], but most were generated with software that did not account for AS (e.g., Newbler prior to version 2.3, CAP3, etc.). Revisiting existing datasets with a cDNA-specific, splice-aware, assembly protocol would provide a far more accurate impression of AS in parasitic nematodes, a factor that can have important practical implications with respect to pathogenesis, drug susceptibility/resistance, vaccine development, etc. [31,32]. For example, the broad-spectrum anthelmintic drug ivermectin is known to bind tightly to one isoform of the α3 glutamate gated chloride channel subunit but not another, and these isoforms appear to be differentially expressed in susceptible versus resistant strains of the cattle parasites *Cooperia oncophora* and *Ostertagia ostertagi*[13,33].

In this study, we used cDNA data from the well-characterized model organism *Caenorhabditis elegans* to define a set of optimized parameters for high-confidence splice isoform prediction using the Newbler assembler. The optimized protocol was then applied to existing and novel cDNA sequences from a diverse array of parasitic nematodes, including *Ancylostoma caninum*, *C. oncophora*, *Dictyocaulus viviparus*, *Necator americanus, Oesophagostomum dentatum*, *Onchocerca flexusoa*, *O. ostertagi*, *Teladorsagia circumcincta*, and *Trichostrongylis colubriformis*[23-30] in the first broad survey of AS among parasitic worms. Our assemblies offer a better impression of genetic and transcriptional complexity in these non-model species and will aid in studies on specific genes/gene families and for annotating and curating draft genomes as they become available.

## Methods

### 454/Roche library construction, sequencing and data cleaning

One splice-leader (SL1) and four oligo(dT) cDNA libraries were constructed from DNase treated *C. elegans* (Bristol N2) RNA according to previously described methods [26]. Libraries were sequenced with a GS 454 FLX pyrosequencer using a standard protocol [34], and raw reads were deposited in the NCBI sequence read archive (SRA) under project number SRP003926. Parasitic nematode sequences were mostly obtained from previous studies, but novel sequences were produced and submitted to the SRA in the same manner (see Additional file 1: Table S1 &[23-30]).

Raw reads were edited and filtered prior to assembly. Relevant adapter sequences were removed with Cutadapt [35], and reads with an overall quality score less than 20 and an overall dust score less than seven were removed using seq_crumbs software (http://bioinf.comav.upv.es/seq_crumbs/). The remaining reads were aligned to rRNA [36,37] and bacterial [38] sequence databases with Bowtie2 (version 2.1.0, default parameters [39]) and to the human (hs37) genome and relevant host genomes with Tophat2 (version 2.0.8, default parameters [40]) for contaminant removal. Host genomes, obtained from GenBank, included: *Canis lupus famliaris* (CanFam3.1) for *A. caninum*; *Bos taurus* (Btau4.6.1) for *C. oncophora*, *D. viviparus*, and *O. ostertagi*; *Sus scrofa* (Sscrofa10.2) for *O. dentatum*; *Ovis aries* (Oar3.1) for *T. colubriformis* and *T. circumcincta. O. flexuosa*, a parasite of European red deer (*Cervus elaphus*), and *N. americanus*, maintained in golden hamsters (*Mesocricetus auratus*), were screened against *Bos taurus* (Btau4.6.1) and the GenBank rodent database (gbrod, downloaded April 24, 2013), respectively, as close substitutes for the unavailable host genomes.

Cleaned *C. elegans* Roche/454 reads were mapped to *C. elegans* coding sequences (WormBase [41] release WS236) with Bowtie2 (version 2.1.0, default parameters [39]) in order to assess the scope of the dataset prior to assembly. The coverage of each feature was assessed using RefCov version 0.3 (http://gmt.genome.wustl.edu/gmt-refcov/) and coverage was reported in Additional file 2: Table S2.

### Assembly and evaluation

Cleaned, decontaminated *C. elegans* Roche/454 reads were assembled into isotigs (transcript isoforms) and clustered into distinct isogroups (putative genetic loci) using the Newbler assembler (version 2.6, mapasm454_source_10142011), invoking the cDNA option. Various combinations of parameters were tested (see Table 1), and the isotigs from each assembly were compared to the *C. elegans* coding sequences (CDSs) and coding transcripts (CDS + UTRs) included in WormBase [41] release WS236 by BLAST + (version 2.2.27) with a cutoff of ≥90% sequence identity over ≥75% the isotig’s length in a single high-scoring segment pair. BLAST + was also used to identify chimeric transcripts with non-overlapping, top BLASTN hits to separate chromosomes or BLASTP matches indicating multiple open reading frames coding in opposite directions. Fragmentation (percentage of matched reference genes with multiple, non-overlapping hits) was calculated from WU-BLAST alignments to CDSs using in-house scripts. To further validate our isoform predictions, Illumina RNAseq libraries were generated from *C. elegans* RNA as previously described [42] (SRA numbers: SRR868958, SRR868932, SRR868957, SRR868939, SRR868942), and the resulting raw reads were mapped to assembled isotigs using Bowtie2 (version 2.1.0, default parameters [39]). The coverage of each isotig, as assessed using RefCov version 0.3 (http://gmt.genome.wustl.edu/gmt-refcov/), is reported in Additional file 3: Table S3.

**Table 1:** ***Caenorhabditis elegans *** **test assemblies**^**1**^

	**cDNA default**	**cDNA -urt**	**cDNA -het**	**cDNA -icl 10**	**cDNA -icl 30**	**cDNA -icl 50**	**cDNA -het -icl 30 -mi 95 -ml 100**
**Assembly statistics**							
% Aligned reads	97.37%	99.09%	97.34%	97.36%	97.37%	97.37%	96.70%
Isotigs	16737	25776	16868	16548	16263	16130	16772
Isotig N50	658	563	658	659	662	662	598
Isogroups	15403	24523	15404	15401	15380	15358	15940
AS Isogroups	824 (5.35%)	823 (3.36%)	824 (5.35%)	802 (5.21%)	741 (4.82%)	674 (4.39%)	691 (4.34%)
Ave. isotigs per AS isogroup	2.62	2.52	2.78	2.43	2.19	2.15	2.20
**Accuracy**							
fragmentation	9.40%	20.70%	9.40%	9.40%	9.40%	9.40%	9.90%
trans-chimeric isotigs^2^	397 (2.37%)	407 (1.58%)	398 (2.36%)	398 (2.40%)	397 (2.44%)	385 (2.38%)	148 (0.88%)
cis-chimeric isotigs^3^	165 (0.99%)	185 (0.72%)	209 (1.24%)	195 (1.18%)	148 (0.91%)	145 (0.90%)	104 (0.62%)
**BLASTN v. CDSs**							
Isotigs with match^4^	6155 (36.77%)	10604 (41.14%)	6158 (36.51%)	6083 (36.76%)	6022 (37.03%)	5996 (37.17%)	6385 (38.07%)
Isogroups with match^5^	5937 (38.54%)	10400 (42.41%)	5940 (38.56%)	5933 (38.52%)	5913 (38.45%)	5901 (38.42%)	6294 (39.49%)
*C. elegans* genes represented	5602 (27.31%)	8470 (41.29%)	5604 (27.32%)	5599 (27.29%)	5583 (27.21%)	5579 (27.19%)	5727 (27.92%)
**BLASTN v. CDS + UTR**							
Isotigs with match^4^	11418 (68.22%)	17031 (66.07%)	11456 (67.92%)	11217 (67.78%)	11053 (67.96%)	10984 (68.10%)	11778 (70.22%)
Isogroups with match^5^	10811 (70.19%)	16512 (67.33%)	10816 (70.22%)	10815 (70.22%)	10789 (70.15%)	10777 (70.17%)	11540 (72.40%)
*C. elegans* genes represented	9600 (46.79%)	12129 (59.12%)	9600 (46.79%)	9598 (46.79%)	9575 (46.67%)	9564 (46.62%)	9748 (47.52%)

^1^Newbler parameters are as follows: urt, include unaligned read tips; het, heterogeneous population; icl, isotig contig length threshold; ml, minimum overlap length; mi, minimum overlap identity.

^2^Trans-chimeric isotigs refer to misassembled transcripts with multiple open reading frames coding in opposite directions.

^3^Cis-chimeric isotigs refer to misassembled transcripts containing sequences derived from distinct regions of the genome assembly.

^4^Matches were required to meet a cutoff of ≥90% nucleotide sequence identity over ≥75% of the length of the isotigs in a single high-scoring segment pair.

^5^Matching isogroups are defined as isogroups containing ≥1 isotig matched to a *C. elegans* feature.

Parasitic nematode transcriptomes were assembled with parameters that showed the optimal performance on *C. elegans* data (minimum overlap of 100 bp and 95% identity, minimum contig length of 30, and heterozygosity specified). Large or complex datasets were reduced to a manageable size by digital read normalization prior to assembly using khmer with a word size of 31 bp (http://ged.msu.edu/papers/2012-diginorm/). *N. americanus* isotigs were compared with transcript isoforms reported along with the genome of *N. americanus*[43] by BLASTN (cutoff of ≥90% sequence identity over ≥75% length of the isotig in a single high-scoring segment pair).

### Annotation of parasitic nematode transcriptomes

Parasite isotigs were searched against the GenBank non-redundant protein database (downloaded July 9, 2013), and non-overlapping top hits with e-value ≤1e-5 were recorded (Additional files 4, 5, 6, 7, 8, 9, 10, 11 and 12: Tables S4-S12). Prot4EST [44] was used to generate translations from the parasite isotigs, and InterPro protein domains and gene ontology terms were predicted from translated proteins using InterProScan [45,46]. Transcript sequences, peptide translations, and annotations are reported in Additional files 4, 5, 6, 7, 8, 9, 10, 11 and 12: Tables S4-S12 and are available from Nematode.net [22].

### Enrichment of AS isogroups associated with functional domains

The number of alternatively spliced and non-alternatively spliced isogroups associated with each InterPro domain was counted (Additional file 13: Table S13), and a non-parametric binomial distribution test was applied to each InterPro domain to test for enrichment of AS isogroups using the following input parameters: (i) the background frequency of AS isogroups across all species (40.5%); (ii) the number of AS isogroups associated with the InterPro domain across all species (i.e., number of “successes”); (iii) the total number of isogroups associated with the InterPro domain across all species (i.e., number of “trials”). In order to reduce false positives resulting from poorly represented domains, domains represented by fewer than ten isogroups or fewer than four species were ignored, reducing total number of domains considered from 5,190 to 3,141 (a 39.5% reduction; Additional file 14: Figure S1). *P* values calculated for each domain were population corrected using False Discovery Rate (FDR) correction [47], and a significance threshold of 0.01 on the corrected *P* values was used to determine which InterPro domains were significantly more often associated with AS isogroups than non-AS isogroups.

## Results and discussion

### Optimization of assembly parameters

**Figure 1:**
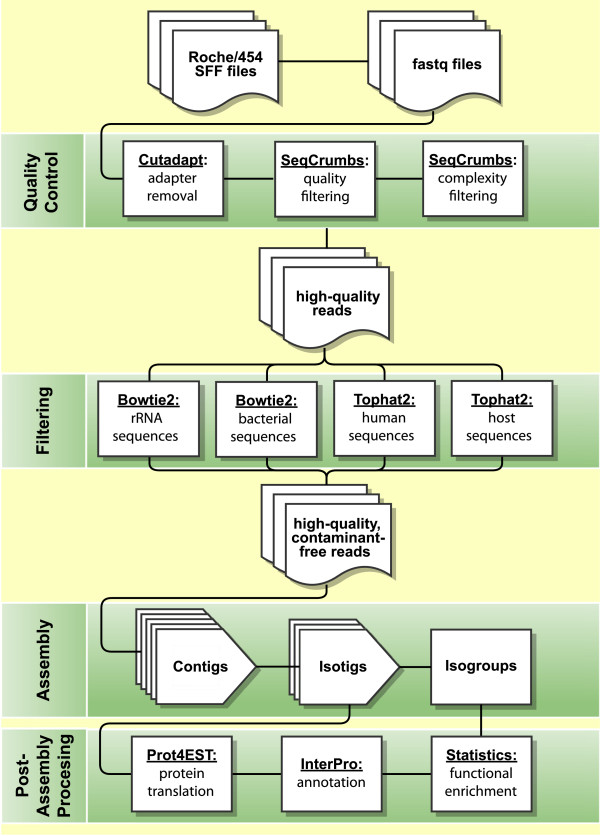
**Roche/454 read processing, decontamination, assembly and annotation.** Raw Roche/454 reads were converted from sff to fastq format for editing and assembly. Relevant adapter sequences were trimmed, and reads failing to meet quality and complexity thresholds were removed. Reads that successfully map to rRNA, bacterial, human or host sequences were also eliminated. The remaining, high-quality, species-specific reads were assembled with Newbler’s cDNA specific protocol using our optimized parameter combination, translated using Prot4EST [44], and annotated using InterProScan [45,46]. Statistical analyses can be carried out at the level of isotigs (unique transcripts) or isogroups (unique genetic loci) depending on the nature of the investigation.

cDNA libraries were generated from mixed stage *C. elegans* worms and sequenced using Roche/454 technology. Our workflow, from the processing of raw reads to the annotation of transcript isoforms and isogroups, is outlined in Figure 1. After trimming, filtering, and contaminant removal, 1,746,642 high-quality reads were mapped to *C. elegans* CDSs to assess the scope of the dataset prior to assembly, and 8,391 CDS isoforms from 7,487 of the 20,515*C. elegans* genes (36.5% of all genes) showed ≥50% breadth of coverage. This level of coverage is comparable to the level seen in previous transcriptomic surveys of non-model organisms [26,28,29] and is sufficient for testing assembly protocols. It was not our intention to perform a thorough study of AS in *C. elegans*; studies of this nature have been reported elsewhere [11].

The Newbler assembler, distributed by 454 Life Sciences, is considered the gold standard for Roche/454 read assembly. Using the cDNA option, Newbler identifies regions of shared sequence, termed contigs, and compiles them into full-length transcripts, termed isotigs. Isotigs with shared contigs, theoretically derived from AS of the same gene, are clustered into isogroups representing distinct genetic loci. We tested various combinations of program parameters in order to reduce assembly errors and increase the percentage of isotigs and isogroups that accurately represent known *C. elegans* sequences (Table 1). The best results were obtained with a contig length of 30 bp, minimum read overlap of 100 bp, minimum sequence identity of 95% (Table 1, last column). The heterozygous mode had little effect on our *C. elegans* assemblies, but we chose to invoke this option to accommodate the genetic heterogeneity of our parasitic worm datasets. Using these parameters, 96.7% of the clean reads were assembled into 15,940 isogroups containing 16,772 isotigs. Some 691 (4.3%) of these isogroups are associated with more than one isotig, with an average of 2.2 isotigs per AS isogroup (Table 1). Approximately 17% of the *C. elegans* genes reported in WormBase build WS236 are associated with more than one CDS isoform with an average of 2.6 isoforms per AS gene [41], and a 2011 study reported that at least 25% of all *C. elegans* genes undergo AS [11]. The relatively low rate of AS detected in our test assembly is probably a reflection of the clonal worm population that, despite being mixed-stage, was dominated at the tissue level by the relatively large adult hermaphrodites. Sampling each sex and life cycle stage independently could have provided greater resolution of AS events; however, the aim of this exercise was to optimize assembly protocols, not to explore AS in the model worm.

By adjusting assembly parameters, we were able to increase the number and percentage of isotigs and isogroups that accurately reflected known CDSs, increase the coverage of the gene set, and reduce the rates of misassembled transcripts (i.e., cis- and trans-chimeras). In the best version of our transcriptome assembly, 38.07% of the isotigs were matched to 7,027 distinct CDS isoforms from 5,727*C. elegans* genes (Table 1, last column). Match rates increased when isotigs were compared to coding transcripts (CDSs plus untranslated regions) rather than CDSs, indicating that a portion of our sequence data corresponds to untranslated regions at the extreme ends of the transcript.

**Figure 2:**
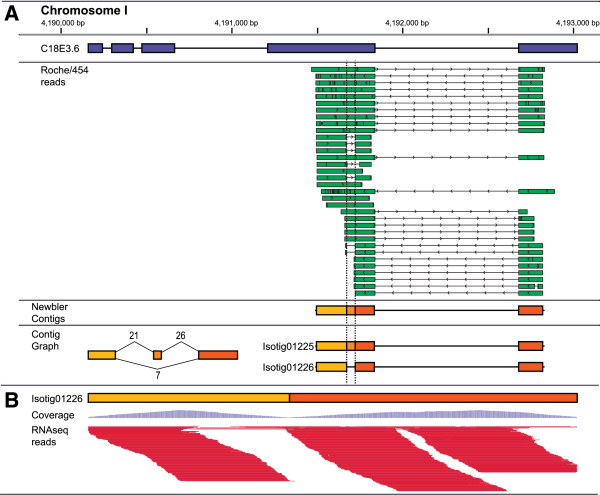
**Alternative splicing of *C. elegans *gene C05B5.5. (A)** Isogroup00600 from our *de novo* cDNA assembly contains two isotigs derived from *C. elegans* gene C18E3.6 (exons depicted as blue bars in top track). Alignment of Roche/454 reads (green bars with arrowheads indicating directionality) gave rise to three distinct contigs (dark, medium and light orange bars). These contigs were pieced together to form isotigs 01225 and 01226 based on read support displayed in the contig graph. Isotig01225, which contains all three contigs, corresponds perfectly to the gene model (blue bars). However, isotig01226 includes only the first (light orange) and third (dark orange), which results in a 50 bp gap with respect to isotig01225 and the gene model. **(B)** Illumina RNAseq reads (dark purple, horizontal bars) mapped to isotig01226 further verifies the junction between the first (light orange) and third (dark orange) contigs, with proportional coverage indicated (light purple, vertical bars). This figure was adapted from alignments visualized using the Integrated Genomics Viewer [48,49].

Despite careful control of assembly parameters, approximately 30% of the assembled isotigs failed to find a BLAST match to a coding transcript isoform (≥90% sequence identity over ≥75% of the length of the isotig in a single high-scoring segment pair). The identification of novel AS isoforms and genes is to be expected given that the reported rates of AS in *C. elegans* have steadily increased over time (Table 2). Therefore, we sought to verify the remaining isotigs using data from another sequencing platform. Of the 4,994 un-matched isotigs, 478 showed 100% breath of coverage with Illumina RNAseq reads, a strong indication that they reflect real, expressed transcript isoforms, not sequence misassembles (see Additional file 3: Table S3). An example of this is illustrated in Figure 2. Isogroup00600 contains two distinct isotigs derived from *C. elegans* gene C18E3.6. One isotig corresponds perfectly to the gene model while the other is missing a 50 base pair segment of the gene’s fourth exon. Sequence reads generated on the Roche/454 and Illumina platforms both support the sequence gap despite the fact that this isoform is not represented in WormBase release WS236.

**Table 2:** ***Caenorhabditis elegans *** **assembly statistics**^**1**^

**Build**	**Date**	**Gene sequences**	**Unique CDS isoforms**
WS150	Oct 2005	20066	20066
WS166	Oct 2006	20082	23207
WS183	Oct 2007	20155	23541
WS196	Oct 2008	20191	23902
WS208	Oct 2009	20238	24202
WS220	Oct 2010	20405	24842
WS228	Oct 2011	20484	25391
WS246	Oct 2012	20537	26041
WS240	Oct 2013	20538	26769

^1^Assemblies and annotations from WormBase [41].

Altogether, 12,265 of the 16,772 isotigs included in our best transcript assembly (73.1%) were verified either by a match to previously reported transcript isoforms included in WormBase or by our orthologous sequencing chemistries. Given the limitations presented by today’s sequencing technologies and assembly software, no combination of parameters will provide a perfect assembly. Some rate of error is to be expected given the challenges presented by complex, dynamic eukaryotic transcriptomes (e.g., varying expression rates, RNA half-life, secondary structure, AS, etc.). However, the error rates we detect are lower than those reported in other studies (particularly those involving shorter reads and deBruijn graph assemblers [50,51]). Clearly, we were able to show improvement over default program parameters using our test dataset, and we expect that the impact of parameter optimization could prove even more vital as the size and complexity of the dataset increases.

The Newbler assembler relies on overlap-layout-consensus (OLC) algorithms for read assembly. These OLC algorithms may be less likely to overestimate the number of isoforms associated with a given gene compared to de Bruijn graph assemblers [18-20,52], but they are computationally intensive and sensitive to the size and complexity of a dataset. Large datasets with many millions of reads from multiple life cycle stages must be reduced prior to assembly. Our optimized protocol performed well with both randomly down-sampled and digitally normalized read sets (Table 3). Interestingly, digital read normalization eliminated nearly half of the reads without much impact on the quality of the assembly, so we have adopted this as our preferred method for dataset reduction prior to assembly.

**Table 3:** **Assembly of down-sampled *Caenorhabditis elegans *** **read sets**

	**Full**	**Random subset**	**Normalized subset**
**Assembly statistics**			
Reads used	1746642	698656	809855
Average read length	403	403	383
% aligned reads	96.70%	94.05%	92.68%
Isotigs	16772	12132	17322
Isotig N50	598	569	599
Isogroups	15940	11746	16129
AS Isogroups	708 (2.65%)	341 (2.90%)	1026 (6.36%)
Average isotigs per AS isogroup	2.20	2.13	2.16
**Accuracy**			
Fragmentation	9.90%	6.30%	9.60%
**BLASTN v. CDSs**			
Isotigs with match^1^	6385 (38.07%)	4424 (36.47%)	6422 (37.07%)
Isogroups with match^2^	6294 (39.49%)	4380 (37.29%)	6224 (38.59%)
*C. elegans* genes matched	5727 (27.92%)	4213 (20.10%)	5628 (27.43%)

^1^Matches were required to meet a cutoff of ≥90% nucleotide sequence identity over ≥75% of the length of the isotigs in a single high-scoring segment pair.

^2^Matching isogroups are defined as isogroups containing ≥1 isotig matched to a *C. elegans* feature.

### Parasitic nematode transcript assemblies

We re-visited previously published Roche/454 data from *C. oncophora*[27], *O. flexuosa*[28], *O. ostertagi*[27], *T. circumcincta*[29], and *T. colubriformis*[25], re-screening and re-assembling with up-to-date, cDNA specific assembly software and our optimized parameters. Additional life cycle stages were sequenced and added to available datasets from *A. caninum*[30], *D. viviparus*[23], *N. americanus*[26], and *O. dentatum*[24] prior to assembly (see Additional file 1: Table S1). Together, these nine species represent a diverse array of parasitic nematodes, in terms of biology as well as phylogeny. *Necator americanus*, one of the two human hookworms, is thought to infect hundreds of millions of people across the Americas, Sub-Saharan Africa and Asia, and is a leading cause of morbidity in children. *A. caninum*, the canine hookworm*,* is an important pathogen in domestic dogs and a model for the study of human hookworm infections. *O. flexuosa* is a filarial nematode and a close relative of *Onchocerca volvulus*, the causative agent of African river blindness. *O. flexuosa* is unique among the Onchocercids in that it is devoid of the bacterial endosymbiont required for development and reproduction in its sister taxa. *D. viviparus*, the bovine lungworm, is the only Trichostrongylid nematode that resides in the lung during its adulthood. *C. oncophora*, *O. dentatum, O. ostertagi*, *T. circumcincta*, and *T. colubriformus* are all intestinal worms of livestock animals and are responsible for significant financial losses in the beef, dairy, sheep, goat and pork industries. Projects have been initiated to sequence the genomes of these species (see Table 4 for BioProject ID numbers), but *N. americanus* is the only species for which a draft genome is presently available [43].

**Table 4:** Parasitic nematode transcript assemblies

	***Ancylostoma caninum***	***Cooperia oncophora***	***Dictyocaulus viviparus***	***Necator americanus***	***Oesophagostomum dentatum***	***Onchocerca flexuosa***	***Ostertagia ostertagi***	***Teladorsagia circumcincta***	***Trichostrongylus colubriformis***
Publication	[30]	[27]	[23]	[26]	[24]	[28]	[27]	[29]	[25]
Genome BioProject ID	PRJNA72585	PRJNA72571	PRJNA72587	PRJNA72135	PRJNA72579	PRJNA230512	PRJNA72577	PRJNA72569	PRJNA74537
Stages	Egg, L1, L2, iL3, aL3, male, female	Egg, L1, L2, iL3, aL3, L4, male, female	Egg, L1, iL3, L5, male, female	iL3, mixed sex adults	L2, iL3, L4, male, female	Mixed sex adults	Egg, L1, L2, iL3, L4, mixed sex adults	Mixed sex adults	Mixed sex adults
Clean reads	4,028,728	6,113,083	4,740,349	1,566,641	2,614,527	1,050,204	7,528,633	1,746,999	2,513,840
Normalized or full assembly	Normalized	Normalized	Normalized	Normalized	Full	Full	Normalized	Full	Full
Number of isotigs	53,978	74,506	50,581	21,320	36,795	22,728	67,599	31,065	37,640
Average isotig length	1,029 bp	763 bp	964 bp	866 bp	815 bp	820 bp	889 bp	989 bp	535 bp
Number of isogroups	35,422	42,785	29,960	16,233	23,061	15,828	37,189	21,780	31,546
Number of AS Isogroups	9,955 (28.10%)	14,180 (33.14%)	10,380 (34.65%)	3,354 (20.66%)	5, 589 (24.24%)	3,869 (24.44%)	11,840 (31.84%)	4,604 (21.14%)	3,686 (11.68%)
Average isotigs per AS isogroup	2.86	3.24	2.99	2.52	3.46	2.78	3.57	3.02	2.65
Number of unique translations	48,713	60,697	44,784	20,286	29,478	20,436	58,022	28,041	35,669
Number of unique InterPro domains	4,103	3,967	4,110	3,823	3,978	2,212	4,903	4,550	3,454
Number of Unique GO terms	1,234	1,211	1,259	1,183	1,239	809	1,428	1,301	1,081

Parasitic nematode transcriptome assembly statistics are reported in Table 4. The datasets range in size and complexity from approximately one million reads derived from adult *O. flexuosa* to upwards of 7.5 million reads derived from eggs, larvae and adults of two geographically distinct strains of *O. ostertagia*. As previously discussed, there is a limit to the amount of data that can be processed by OLC assembly algorithms like those implemented by Newbler, so several datasets had to be reduced by digital read normalization prior to assembly (Table 4). Our tests seem to indicate that the complexity of the transcriptome has a greater impact on assembly efficiency than the absolute number of reads. For instance, we were able to assemble some 2.5 million reads from mixed sex adult *T. colubriformis*, whereas a full assembly of the 1.5 million reads derived from L3 and mixed sex adult *N. americanus* was not possible.

The number of isogroups obtained from each assembly ranged from 15,828 from *O. flexuosa* to 42,785 from the more thoroughly covered transcriptome of *C. oncophora*. Detected rates of AS, as measured by the number of isogroups associated with multiple isotigs, mostly fell within the 20-30% range, with a maximum AS rate of 34.65% in *D. viviparus* (Table 4). The AS rates seen in the parasitic nematodes were expected to be similar, as previous studies have shown that splice events are highly conserved among *Caenorhabditis* species despite hundreds of millions of years of evolutionary separation [53,54]. It is also reasonable to expect that the parasitic nematodes, especially those with extremely complex life cycles like *N. americanus* and *D. viviparus*, would have higher rates of AS than free-living worms like *C. elegans* due to the increased genomic complexity that may be required to interact with multiple hosts/vectors, host/vector tissues, and environmental conditions. We did not make an effort to classify or compare the nature of these AS events (e.g., alternative starts and/or stops, intron retention, exon skipping, etc.), but we expect that this will be possible in future studies aimed at exploring AS profiles of particular species in greater detail.

It stands to reason that sampling and sequencing more life cycle stages would lead to increased resolution of AS events. Indeed, including more stages tended to increase the number of isogroups (i.e., genetic loci) identified, but overall AS rates and the average number of isotigs associated with each isogroup remained relatively consistent with the notable exception of *T. colubriformis*. The AS rate reported for *T. colubriformis* (11.68%) was much lower than AS rates reported for other species represented by a single cDNA library derived from mixed-sex adults (24.44% AS in *O. flexuosa* and 21.14% AS in *T. circumcincta*). This disparity may be due to decreased transcriptomic complexity in *T. colubriformis*, but there may be other explanations. In the case of *T. colubriformis*, material was obtained from an inbred laboratory strain [25], while *O. flexuosa* and *T. circumcinta* material were collected in the field [28,29]. *O. flexuosa* nodules tend to be dominated by large, adult females [55]. Likewise, *T. circumcinta* is a polymorphic species with sex ratios biased towards females [56]. This is significant due to the fact that a patent female represents a broad survey of adult female tissues, embryos in various stages of development, and even stored sperm from males, all of which contribute to diversity in the transcript population. Sequencing additional life cycle stages of *T. colubriformis* or specifically studying adults of other species would provide additional data needed to better understand the obtained results.

The assembled sequences from these nine species, as well as their functional annotations and predicted translations are available from Nematode.net [22] for use by the wider community. Although genome sequencing projects are currently underway, the transcriptomes presented here represent a significant proportion of the sequence data available from these species at this time. These datasets are, therefore, a vital source of information on the genetic content and complexity of these parasites and will remain so even after draft genomes are published as genome sequencing does not, in and of itself, provide any information on AS. Historically, initial reports of draft genomes rarely comment on AS [57-64]. For instance, the draft genome of the well-studied filarial nematode *B. malayi* was published in 2007, but the report made no mention of AS [59]. The most recent dataset available from WormBase (*B. malayi* WS236) includes multiple isoforms for 16% of the reported genes, but no comprehensive studies on the subject of AS in *B. malayi* have been reported despite an abundance of representative RNAseq data [65]. The recently published *N. americanus* genome paper was unique in that it included an estimate of AS based on Illumina RNAseq data generated from L3 and adult worms. Multiple isoforms were identified from approximately 25% of the 19,151 predicted protein coding genes. Some 1,209 of the 3,354 AS isogroups from *N. americanus* match 1,114 genes reported as AS in the genome study (≥90% nucleotide sequence identity over ≥75% of the length of the isotigs in a single high-scoring segment pair) [43], while another 65 AS isogroups matched genes that previously lacked evidence for AS. Clearly our assemblies, performed with a special emphasis on AS, will be a useful complement to genome sequencing studies and transcriptome studies performed using orthologous sequencing and assembly approaches.

### Protein domains associated with alternative splicing

Given the fact that in AS, both the patterns of splicing as well as the spliced exons themselves, tend to be evolutionarily conserved [53,54], we wanted to explore potential links between AS and genetic function. Coding sequences were predicted for each of the transcript assemblies and these were searched for similarity to InterPro protein domains. A total of 5,692 unique InterPro domains were identified from all species included in this study, with counts ranging from 4,904 domains in *O. ostertagi* to 2,212 in *O. flexuosa* (Table 4). Some 40.5% of all isogroups associated with an InterPro domain are AS, and 21 InterPro protein domains were significantly correlated with AS isogroups (Table 5). Functions related to nucleotide binding are prevalent in this list. Nucleic acid binding proteins have a wide variety of functions, localization patterns, and binding preferences that can certainly be affected by AS [66-68]. For example, AS of the UNC-62 transcription factor produces two distinct isoforms in *C. elegans*. These isoforms localize to different tissues, exhibit different temporal expression patterns, and seem to bind different DNA consensus sequences due to alterations in the DNA binding domain [69,70]. Chromo (CHRomatin Organization Modifier) and chromo-like domains interact with histones and nucleic acids, and studies have shown that AS of these proteins can have major implications on function, which, in turn have implications on gene expression and organismal development [71]. Future studies will be required to further explore the link between AS and protein function in parasitic nematodes, as well as to elucidate its specific biological consequences.

**Table 5:** Enrichment of InterPro protein domains among alternatively spliced isogroups

**InterPro protein domain**		**Number of species with domain**	**Total number of Isogroups containing domain**	Percentage of isogroups with domain that are AS*	P value for enrichment**
IPR000504	RNA recognition motif domain	9	858	50.7%	2.1E-06
IPR016197	Chromo domain-like	9	128	64.1%	3.8E-05
IPR012677	Nucleotide-binding, alpha-beta plait	9	1037	48.7%	4.2E-05
IPR006092	Acyl-CoA dehydrogenase, N-terminal	8	53	73.6%	2.0E-04
IPR013786	Acyl-CoA dehydrogenase/oxidase, N-terminal	9	68	69.1%	3.2E-04
IPR003593	AAA + ATPase domain	8	189	57.7%	3.6E-04
IPR001412	Aminoacyl-tRNA synthetase, class I, conserved site	8	43	74.4%	6.6E-04
IPR006091	Acyl-CoA oxidase/dehydrogenase, central domain	9	70	67.1%	7.6E-04
IPR009100	Acyl-CoA dehydrogenase/oxidase, N-terminal and middle domain	9	93	63.4%	8.9E-04
IPR011993	Pleckstrin homology-like domain	9	474	50.4%	1.7E-03
IPR014001	Helicase, superfamily 1/2, ATP-binding domain	9	317	52.1%	3.7E-03
IPR023780	Chromo domain	9	79	63.3%	3.6E-03
IPR000953	Chromo domain/shadow	9	85	62.4%	3.7E-03
IPR015421	Pyridoxal phosphate-dependent transferase, major region, subdomain 1	9	275	52.7%	3.8E-03
IPR002194	Chaperonin TCP-1, conserved site	8	55	67.3%	3.6E-03
IPR003954	RNA recognition motif domain, eukaryote	9	41	70.7%	4.5E-03
IPR006020	PTB/PI domain	9	71	63.4%	5.7E-03
IPR001650	Helicase, C-terminal	9	298	51.7%	6.8E-03
IPR017998	Chaperone tailless complex polypeptide 1 (TCP-1)	9	66	63.6%	7.6E-03
IPR011545	DNA/RNA helicase, DEAD/DEAH box type, N-terminal	9	275	52.0%	7.3E-03
IPR002495	Glycosyl transferase, family 8	7	11	90.9%	7.2E-03

*In total, 40.5% of all isogroups associated with any InterPro domain are AS.

**Binomial test, FDR corrected, threshold value of 0.01.

## Conclusions

*De novo* transcriptome assembly is a complicated procedure that is confounded by varied gene expression patterns, such AS of mRNA. Transcriptome assemblies benefit from the use of optimized parameters designed to increase accurate coverage of the gene set and minimize assembly error. The set of parameters we described was thoroughly tested with *C. elegans* data and verified using well-curated sequences available from WormBase as well as data from an unrelated sequencing chemistry. Our optimized parameters are offered as a guide to assist in the assembly of other nematode transcriptomes, and updated, annotated transcript assemblies from nine species of parasitic worms are offered as a resource to the research community. Rates of AS seem to be similar among the species studied, and 21 InterPro protein domains appear to be enriched among AS transcripts. This represents a first step in exploring AS among parasitic nematodes, an important and relevant topic that should be further investigated in future sequencing studies.

## Competing interests

The authors declare that they have no competing interests.

## Authors’ contributions

SA and MM conceived the study. SA and SNM processed and assembled *C. elegans* cDNA data and tested the assemblies. SNM processed and assembled parasitic nematode cDNA data and annotated the parasitic nematode transcriptomes. BAR performed statistical analyses. SA, SNM and MM drafted the manuscript. All authors read and approved the final version of the manuscript.

## Supplementary Material

Additional file 1: Table S1 454/Roche sequencing of parasitic nematode transcriptomes. Click here for file

Additional file 2: Table S2 Coverage of *Caenorhabditis elegans* CDSs with Roche/454 reads. Click here for file

Additional file 3: Table S3 Coverage of assembled *Caenorhabditis elegans* isotigs with Illumina RNAseq reads. Click here for file

Additional file 4: Table S4 The annotated transcriptome of *Ancylostoma caninum.* Click here for file

Additional file 5: Table S5 The annotated transcriptome of *Cooperia oncophora.* Click here for file

Additional file 6: Table S6 The annotated transcriptome of *Dictyocaulus viviparus.* Click here for file

Additional file 7: Table S7 The annotated transcriptome of *Necator americanus.* Click here for file

Additional file 8: Table S8 The annotated transcriptome of *Oesophagostomum dentatum.* Click here for file

Additional file 9: Table S9 The annotated transcriptome of *Onchocerca flexuosa.* Click here for file

Additional file 10: Table S10 The annotated transcriptome of *Ostertagia ostertagi.* Click here for file

Additional file 11: Table S11 The annotated transcriptome of *Teladorsagia circumcincta.* Click here for file

Additional file 12: Table S12 The annotated transcriptome of *Trichostrongylus colubriformis.* Click here for file

Additional file 13: Table S13 Alternatively spliced and non-alternatively spliced isogroups associated with InterPro protein domains. Click here for file

Additional file 14: Figure S1 Representation of InterPro protein domains among parasitic nematode species and isogroups. The plot indicates the total number of isogroups (from all species) associated and parasitic nematode species associated with a given InterPro protein domain. In order to reduce false positives resulting from poorly represented domains, InterPro domains represented by fewer than ten isogroups and/or fewer than four species were excluded from enrichment analyses. Red lines indicate cutoff values. Click here for file
