# Supplementary material for: Identification and characterization of alternative splicing in parasitic nematode transcriptomes
Source: Parasit Vectors. 2014 Apr 1;7:151. doi: 10.1186/1756-3305-7-151 (PMC3997825; doi:10.1186/1756-3305-7-151)
Supplement: Additional file 1: Table S1 — 454/Roche sequencing of parasitic nematode transcriptomes. [file 1756-3305-7-151-S1.docx]

**Table S1. 454/Roche sequencing of parasitic nematode transcriptomes**

| **Species** | **Stage** | **454/Roche Chemistry** | **Novel or Published** | **Citation or**  **SRA Experiment Accession(s)** |
| --- | --- | --- | --- | --- |
| *Ancylostoma caninum* | egg |  |  | SRX092626 |
|  | L1 | Titanium FLX | novel | SRX092673 |
|  | L2  activated L3 |  |  | SRX092675  SRX092674 |
|  | infective L3 activated L3 adult male adult female | GS FLX | published | [Wang, Abubucker et al. (2010](#_ENREF_8)) |
|  |  |  |  |  |
|  |  |  |  |  |
|  |  |  |  |  |
| *Cooperia oncophora* | egg L1 L2 infective L3 activated L3 L4 adult male adult female | Titanium FLX | published | [Heizer, Zarlenga et al. (2013](#_ENREF_5)) |
|  |  |  |  |  |
|  |  |  |  |  |
|  |  |  |  |  |
|  |  |  |  |  |
|  |  |  |  |  |
|  |  |  |  |  |
|  |  |  |  |  |
| *Dictyocaulus viviparus* | egg |  |  | SRX100630, SRX092678 |
|  | mixed L1/L2 | Titanium FLX | novel | SRX092676 |
|  | L5 |  |  | SRX100629 |
|  | infective L3 adult male adult female | Titanium FLX | published | [Cantacessi, Gasser et al. (2011](#_ENREF_1)) |
|  |  |  |  |  |
|  |  |  |  |  |
| *Necator americanus* | infective L3 | Titanium FLX | novel | SRX202019 |
|  | mixed sex adults | Titanium FLX | published | [Cantacessi, Mitreva et al. (2010](#_ENREF_4)) |
| *Oesophagostomum dentatum* | L2 | Titanium FLX | novel | SRX092679 |
|  | L3 L4 male female | Titanium FLX | published | [Cantacessi, Jex et al. (2010](#_ENREF_2)) |
|  |  |  |  |  |
|  |  |  |  |  |
|  |  |  |  |  |
| *Onchocerca flexuosa* | mixed sex adults | Titanium FLX | published | [McNulty, Abubucker et al. (2012](#_ENREF_6)) |
| *Ostertagia ostertagi,* Beltsville | infective L3 activated L3 L4 mixed sex adults | Titanium FLX | published | [Heizer, Zarlenga et al. (2013](#_ENREF_5)) |
|  |  |  |  |  |
|  |  |  |  |  |
|  |  |  |  |  |
| *Ostertagia ostertagi,* Ghent | Egg L1 L2 | Titanium FLX | published | [Heizer, Zarlenga et al. (2013](#_ENREF_5)) |
|  |  |  |  |  |
|  |  |  |  |  |
| *Teladorsagia circumcincta* | mixed sex adults | Titanium FLX | published | [Menon, Gasser et al. (2012](#_ENREF_7)) |
| *Trichostrongylus colubriformis* | mixed sex adults | GS FLX | published | [Cantacessi, Mitreva et al. (2010](#_ENREF_3)) |

**References:**

Cantacessi, C., R. B. Gasser, et al. (2011). "Deep insights into Dictyocaulus viviparus transcriptomes provides unique prospects for new drug targets and disease intervention." Biotechnol Adv **29**(3): 261-271.

Cantacessi, C., A. R. Jex, et al. (2010). "A practical, bioinformatic workflow system for large data sets generated by next-generation sequencing." Nucleic Acids Res **38**(17): e171.

Cantacessi, C., M. Mitreva, et al. (2010). "First transcriptomic analysis of the economically important parasitic nematode, Trichostrongylus colubriformis, using a next-generation sequencing approach." Infect Genet Evol **10**(8): 1199-1207.

Cantacessi, C., M. Mitreva, et al. (2010). "Massively parallel sequencing and analysis of the Necator americanus transcriptome." PLoS Negl Trop Dis **4**(5): e684.

Heizer, E., D. S. Zarlenga, et al. (2013). "Transcriptome analyses reveal protein and domain families that delineate stage-related development in the economically important parasitic nematodes, Ostertagia ostertagi and Cooperia oncophora." BMC Genomics **14**: 118.

McNulty, S. N., S. Abubucker, et al. (2012). "Transcriptomic and proteomic analyses of a Wolbachia-free filarial parasite provide evidence of trans-kingdom horizontal gene transfer." PLoS One **7**(9): e45777.

Menon, R., R. B. Gasser, et al. (2012). "An analysis of the transcriptome of Teladorsagia circumcincta: its biological and biotechnological implications." BMC Genomics **13 Suppl 7**: S10.

Wang, Z., S. Abubucker, et al. (2010). "Characterizing Ancylostoma caninum transcriptome and exploring nematode parasitic adaptation." BMC Genomics **11**: 307.
