# Supplementary figures and images for: Identification and characterization of alternative splicing in parasitic nematode transcriptomes
Source: Parasit Vectors. 2014 Apr 1;7:151. doi: 10.1186/1756-3305-7-151 (PMC3997825; doi:10.1186/1756-3305-7-151)

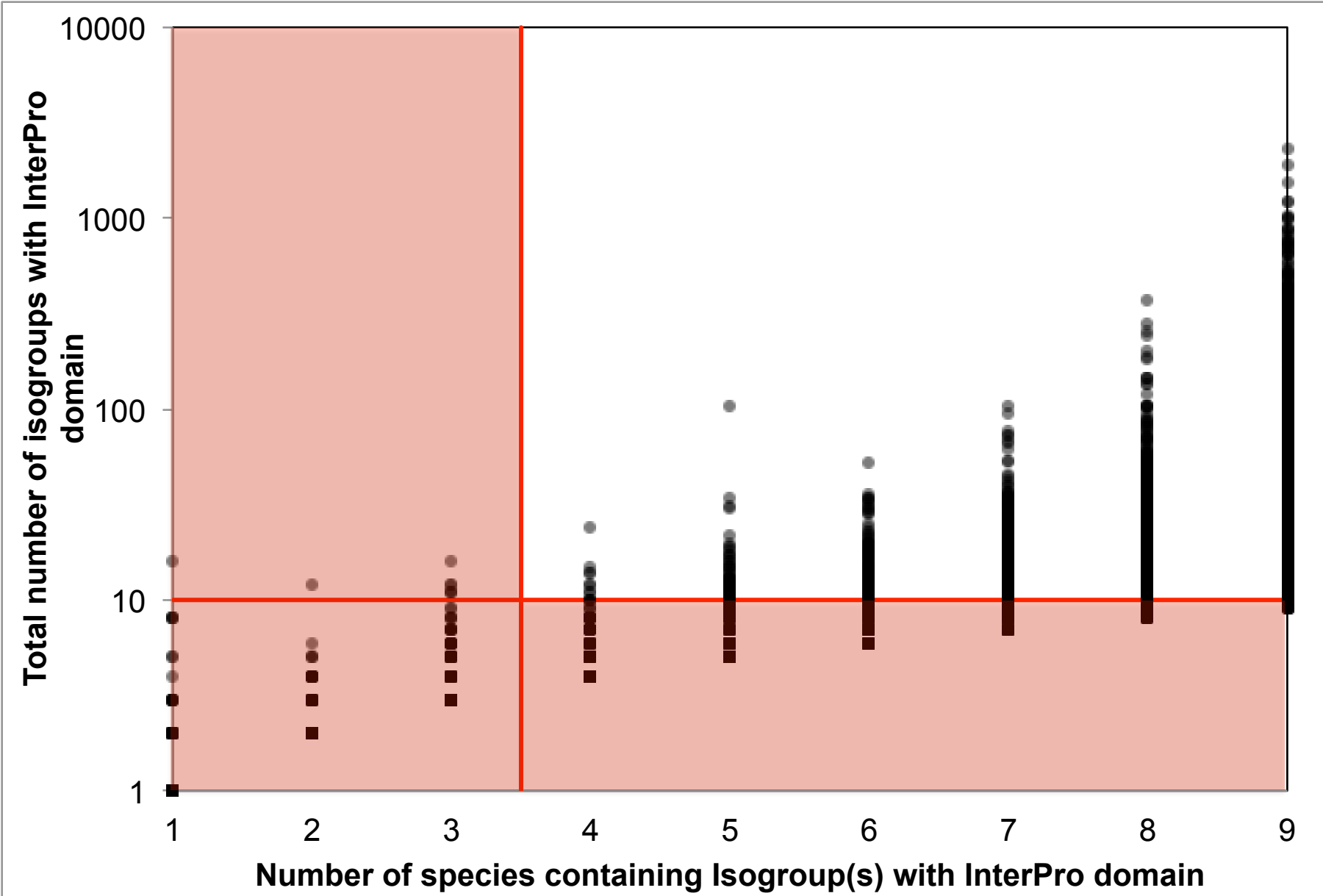

Supplement: Additional file 14: Figure S1 — Representation of InterPro protein domains among parasitic nematode species and isogroups. The plot indicates the total number of isogroups (from all species) associated and parasitic nematode species associated with a given InterPro protein domain. In order to reduce false positives resulting from poorly represented domains, InterPro domains represented by fewer than ten isogroups and/or fewer than four species were excluded from enrichment analyses. Red lines indicate cutoff values. [file 1756-3305-7-151-S14.pdf]
